# PhInd—Database on Polyphenol Content in Agri-Food By-Products and Waste: Features of the Database

**DOI:** 10.3390/antiox13010097

**Published:** 2024-01-12

**Authors:** Nemanja Teslić, Milica Pojić, Alena Stupar, Anamarija Mandić, Branimir Pavlić, Aleksandra Mišan

**Affiliations:** 1 Institute of Food Technology, University of Novi Sad, Bulevar Cara Lazara 1, 21000 Novi Sad, Serbia; milica.pojic@fins.uns.ac.rs (M.P.); alena.tomsik@fins.uns.ac.rs (A.S.); anamarija.mandic@fins.uns.ac.rs (A.M.); aleksandra.misan@fins.uns.ac.rs (A.M.); 2 Faculty of Technology, University of Novi Sad, Bulevar Cara Lazara 1, 21000 Novi Sad, Serbia; bpavlic@uns.ac.rs

**Keywords:** extraction technology, plants, bioactive compounds, valorization, HPLC

## Abstract

Timely access to topic-relevant datasets is of paramount importance for the development of any successful strategy (food waste reduction strategy), since datasets illuminate opportunities, challenges and development paths. PhInd is the first comprehensive database on polyphenol content in plant-based by-products from the agri-food sector or the wastewater sector and was developed using peer-reviewed papers published in the period of 2015–2021. In total, >450 scientific manuscripts and >6000 compound entries were included. Database inclusion criteria were polyphenol contents = determined using HPLC/UHPLC quantitative methods. PhInd can be explored through several criteria which are either ‘open’ or checkboxes. Criteria are given in subsections: (a) plant source; (b) by-product industrial processing; (c) pre-treatment of by-products before the isolation of polyphenols; and (d) the extraction step of polyphenols. Database search results could be explored on the website directly or by downloading Excel files and graphs. This unique database content is beneficial to stakeholders—the food industry, academia, government and citizens.

## 1. Introduction

Climate change, loss of biodiversity and the degradation of the environment are seriously posing threats to life on Earth. In 2019, the agri-food system’s greenhouse gas emissions were estimated to be 16.5 billion metric tons, corresponding to 31% of total anthropogenic emissions [1]. Food is wasted throughout the entire food supply chain from farms to the household level, and there is a need to develop strategies to reduce food waste by at least 30% of all discarded food waste by the end of 2025 [2]. As part of the food system, the processing of plant raw materials in food industries generates a significant amount of non-edible by-products (shells, skins, stems, seeds and pulps) which are costly to dispose in landfills or incinerators, causing greenhouse gas emissions [3], even though these by-products could be used for feed or for added value food products as a source of dietary fiber or bioactive compounds [4]. Apart from them, solid waste from the aromatic plant essential oil industry is a rich source of bioactive compounds for the food and pharmaceutical industry, which at this moment is not valorized [5]. Waste/processing water from fruit or vegetable industries may also contain a significant amount of polyphenols, e.g., olive processing [6] or artichoke blanching [7], which could be further used as a cheap raw material for the nutraceutical and pharmaceutical industry. Fruit and vegetable processing by-products, apart from basic nutrients, polysaccharides, lipids and proteins, possess added-value compounds with high functionality and/or bioactivity which can be used to produce food additives and products for therapeutic purposes [8]. In line with the Green Deal and “farm-to-fork” action plans, there is a need for innovation in the use and acquisition of high value-added compounds from agri-food wastes and by-products. The circular biobased economy is still a largely untapped potential for the development of sustainable businesses.

Polyphenols are a large group of plant non-nutrient natural products, plant secondary metabolites that possess a broad spectrum of biological activities such as antioxidant, antimicrobial, anticancer, anti-inflammatory, immunomodulatory, antiaging, hepatoprotective, enzyme inhibitory and antidiabetic [9]. It is well documented that the consumption of plants, fruits and vegetables is associated with a healthy diet because plants, in addition to the variety of nutrients, present the main source of plant phenolics. To provide detailed and comprehensive information on the nature and quantities of polyphenols found in the main foods consumed with the diet, the USDA (United States Department of Agriculture) has published several Special Interest Databases (SID) on flavonoids (version 2.1. released in 2018), isoflavones (version 2.1. released in 2015) and pro-anthocyanidins (version 3.3. released in 2018) content [10]. In addition to that, a Phenol-Explorer web-based database on polyphenol content in foods has been developed to offer information on the polyphenols present in a given food, the dietary sources of a given polyphenol and the quantity of a specific compound found in a given food [11]. Apart from the well-known health benefits of polyphenolic-rich diets, adverse outcomes have been reported upon consumption of polyphenolic drinks among persons with thyroid disease, high blood pressure, degenerative disease, epilepsy and heart disease. These data are collected in a dynamic web-based database (ToxDP2) to assess dietary polyphenols’ safety and fulfill the toxicity data gaps in the domain of food safety [12].

Plant-based extracts enriched with polyphenols have been extensively used by the food and cosmetic industry [4]. The amount of extracted polyphenols was estimated to be 33,880 tons in 2024, valued at USD 1.33 billion because of the growing demand for polyphenol-rich products [13]. There is also a large number of papers and patents dealing with the extraction, analysis and testing of plant phenolics from fruit, vegetable and medicinal plant processing by-products; however, the degree of commercialization of these results is low. Possible reasons are as follows: the existing data are not systematized; there are no standardized methods for the analysis of polyphenolic compounds; there is a large number of polyphenolic compounds; there is variation in the content of polyphenolic compounds depending on the species, variety, year, origin, pre-treatment, treatment, type of extraction, etc.

Referring to all of the abovementioned, the aim of this work is to present a database—PhInd—that collects and displays the data on the content of polyphenols in agri-food waste and by-products (http://phind.uns.ac.rs/ accessed on 13 December 2023). We were guided by the idea to collect and systematize the available data as best and precisely as possible so that the desired information would be easily accessible through the use of defined search criteria. Apart from the type of agri-food waste and by-products and its botanical origin, the source of agri-food waste and by-products (e.g., branch of industry, industrial process and scale of process), total polyphenol content, information about extraction methods, pre-treatments applied to assist the extraction (drying, defeating, hydrolysis and application of assisting technologies), extraction solvents and techniques used, the content of individual polyphenol compounds is provided, which can help researchers to discover trends, identify correlations and monitor the research gaps. Industry can utilize the database for new product development, process optimization, and exploration of new opportunities and ideally the creation of new value chains. PhInd can help consumers make smart choices and understand the benefits of consuming polyphenol-rich foods and reducing food waste in households. Last but not least, public authorities and political decision makers could utilize PhInd as a tool for the development of action plans and strategies to meet the aimed food loss reduction. Practically, this unique database content is beneficial to all stakeholders involved in the quadruple helix innovation framework (industry, academia, government and citizens).

## 2. Materials and Methods

### 2.1. Construction of the Database

The PhInd database was developed using PHP as the programming language and CakePHP as the web framework, with MySQL serving as the chosen database system (https://www.mysql.com/ accessed on 13 December 2023). The PhInd website is publicly available at https://phind.uns.ac.rs/ accessed on 13 December 2023. There are no restrictions regarding the web browsing software used; it can be viewed on different versions of Safari, Google Chrome and Firefox. The development of the PhInd database encompassed five key stages: literature search, data compilation, data evaluation, data aggregation and the ultimate export of data to the MySQL database.

### 2.2. Database Content

When searching for scientific literature from which to select data for inclusion in the database, the following keywords were used: by-product AND polyphenol OR phenolic OR anthocyanin OR flavan-3-ol OR flavanol OR flavonol OR valorization. Scientific literature was searched through Scopus, Google Scholar, Science Direct and Web of Science portals. The inclusion criteria employed during data selection for the database required the polyphenol content to be determined using HPLC/UHPLC semi-quantitative and quantitative methods, while HPLC/UPHLC qualitative methods were not included in PhInd. The database development was primarily reliant on peer-reviewed full-text papers addressing food/agricultural by-products of plant origin published between 2015 and 2021, containing a total of >450 scientific publications, 690 entries and >6000 composition data. The database contains information on individual dietary polyphenols found in agricultural waste and food by-products. This information includes specific background details such as the type and source of the waste/by-products, the plant part used, the type of industry, and the industrial processing step responsible for generating the waste, as well as the link to the manuscript with “raw” data. Additionally, it provides specific details about the polyphenol extraction process, including sample pre-treatment, extraction techniques and solvents used. To ensure data accuracy, a double-verification process was employed for all entered information, while an external review process was applied to further improve PhInd features. Different individuals cross-checked the data that they did not input themselves following established validation guidelines. Any discrepancies or errors identified during this verification process were corrected to maintain the integrity of the database. Before explaining in detail all features of the PhInd database, it is necessary to make a disclaimer stating that there are definitely more eligible scientific manuscripts in the literature for this database; however, due to a lack of time, “only” 690 entries in total were included, while many were not included due to the lack of HPLC/UHPLC polyphenols quantification data. We believe that even this partial, yet extensive and up-to-date, coverage of the literature on polyphenol content in agri-food by-products would provide valuable information for various stakeholders.

## 3. Results and Discussion

### 3.1. Main Page Interface and Subsections

**Figure 1:**
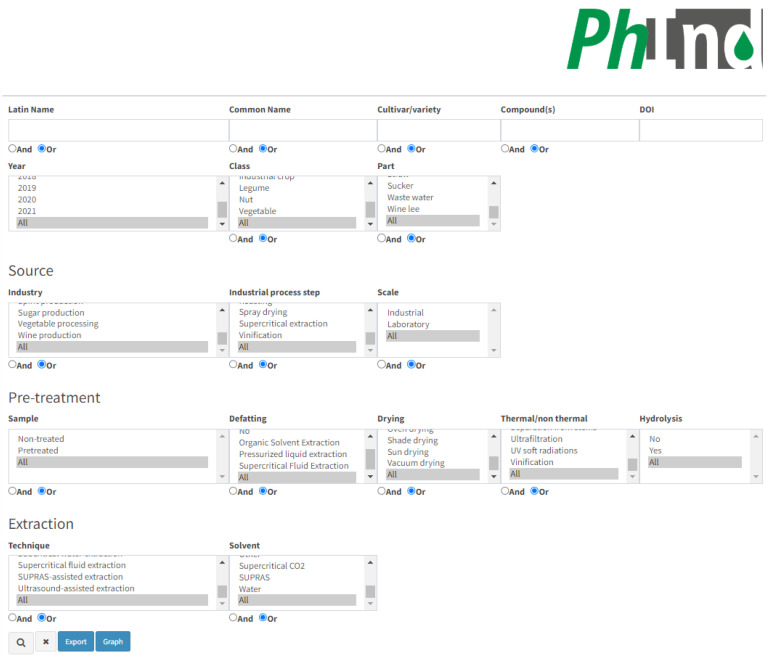
Search interface of the PhInd database.

PhInd is a comprehensive database on the polyphenol content in by-products or waste generated by food processing or harvesting. This database was developed with a web interface which should provide a user-friendly search based on one or multiple criteria. A screenshot of the main PhInd search interface e is given in Figure 1, while the list of the generated results is given below that.

The database can be explored through several criteria which are either ‘open’ or can be selected as checkboxes. They are given in four different subsections which are related to (a) plant material properties, compounds and manuscript identity-related information; (b) source properties related to industrial processing; (c) pre-treatment of the by-product prior to the isolation of bioactive compounds; and (d) the extraction step applied for the isolation of polyphenols. The first subsection contains several ‘open’ fields, while others consist of checkboxes only.

Plant material, e.g., resource, could be explored by three ‘open’ fields which are related to “Latin name”, “Common name” and “Cultivar/variety”. PhInd covers a wide range of by-products or wastes from different plant species, including fruits (e.g., cranberry, lingonberry, açaí, apple, grape, olive, etc.), herbs (basil, fennel, sage, lavender, etc.), cereals (e.g., barley, wheat, rice, corn, etc.), legumes (e.g., lentil, carob, sweet lupin, etc.), nuts (almond, hazelnut, coconut, chestnut, etc.), industrial crops (cotton, rapeseed, soya, sugarcane, etc.) and vegetables (potato, artichoke, broccoli, beetroot, etc.). It should be highlighted that we tried to be as consistent as possible with the insertion of data in the database. That means that we relied particularly on the exact information given by the authors of the paper which was cited. For example, orange can be found by several different Latin names, depending on the work which was cited: *Citrus × sinensis*, *Citrus sinensis* or *Citrus sinensis* L. Therefore, we strongly advise readers to explore several different search options in order to broaden or narrow their search. For example, typing only “citrus” in the Latin name field will result in listing all resources which have the word “citrus” in the Latin name of the species. On the other hand, users could be more specific and search for “*Citrus aurantium*”, which will result only in hits associated with the bitter orange. Cultivar/variety information is missing for some entries (inserted as not applicable—N.A.) as it also depends on the information given in the original manuscripts. Also, not all cultivars from each manuscript were included in the database. For example, out of 44 grape seed samples in total, only cv. Büzgülü (Gülnar) was included in the database as the sample with the highest total phenolic content (spectrophotometry) and cv. Ekşikara as the sample with the highest total flavonoid content (spectrophotometry) [14]. Only peach cv. Sweet cap was included out of 5 cultivars as the sample with the highest total phenolic content, etc. [15]. Plant resources could be also explored by the entire “Class” (e.g., cereal, fruit, herb, etc.) and “Part” (e.g., leaf, seed, root, pomace, etc.) checkbox lists. “Part” checkbox terms included in PhInd could be divided into two big groups: (1) by-products related to plant parts such as seed [16], leaf [17], stem [18], peel [19], etc.; (2) specific food industry by-products such as sugar molasses [20], olive pâté [21], wine lee [22], wastewater [6], various pressed cakes [23], etc. Thus, these individual terms (or any other terms) could be added to narrow down the search toward more specific targets. For example, searching for the Apple in the “Common name” field provides forty different hits, while an additional selection of the Skin from the “Part” checkbox narrows down the search to only one result. Similarly, as for cultivars, not all samples were included in the database, e.g., only one sample of peach young leaves, mature leaves and stems was added out of five samples for each plant part [15]. Furthermore, if the experimental trial of a specific entry included a mixture of two parts listed in the checkbox “Part”, both plant parts were included in that entry, e.g., bark and seed of acerola and guava [24]. If three or more plant parts were mixed and used for polyphenols extraction, e.g., onion edible outer dry layers and apical and basal trimmings, the plant part in this entry was marked as mix [25]. There are also some specific mixtures which are often found in the literature such as fruit pomaces consisting of skin, seeds and residual pulp which are marked as pomace due to high frequency in the literature [26,27]. It is also possible to select the term “All” in “Class” and “Part” checkboxes (this is applicable for all checkboxes) which will enable the selection of all plant classes and parts added in PhInd. As it was mentioned above, we relied particularly on the exact information given by the authors of the paper which was cited; thus, apple fruit outer part was inserted in PhInd as peel [28] or skin [29], depending on the manuscript. This section could be also explored according to the information of the manuscript (Digital Object Identifier—DOI and publication year) from which the results were added in the database, enabling the narrowing down of the search to specific publications or focusing on papers published within a particular time frame. Furthermore, we advise the authors exploring this topic who published similar research in this field from 2015–2021 to explore whether their work could be found in the PhInd database. Lastly, this section could be also explored by ‘open’ field “Compound(s)”, which aims to make easier the search focused on the target polyphenols that could be isolated from the various side streams and by-products. This feature is particularly useful for researchers interested in studying the chemical composition of plants or exploring the presence of specific compounds in different plant species, or for nutraceutical, cosmetic and pharmaceutical industries interested in finding alternative sources of valuable polyphenols. Since some compounds are added to PhInd in various forms and synonyms, users are advised to search specific compounds in all variations. For example, in addition to the term chlorogenic acid [30], this compound could be searched as 5-O-caffeoylquinic acid [31] and 5-caffeoylquinic acid [32]. Rutin [33] can be searched also as quercetin-3-O-rutinoside [34], resveratrol [35] as cis- or trans-resveratrol [36], etc. There are also some less specified polyphenols such are hydroxycinnamic acid derivative, phenolic acid derivative [37], caffeic acid derivative [38], taxifolin derivatives [39], etc.

The second section aims to provide more information about the industrial processing of the material used for the extraction of polyphenols. It consists of three checkbox lists: “Industry”, “Industrial process step” and “Scale”. The checkbox “Industry” aims to improve search options toward various industrial branches in general (e.g., agricultural waste, fruit processing, vegetable processing, etc.). A more specific search could be performed with the addition of “Industrial process step” (e.g., blanching, brewing, fermentation, roasting, and more), aiming to identify the most distinct processing step/unit operation used in industrial processing which generated a particular by-product. “Industrial process step” stands for industrial processes which were performed in the industry to produce by-products and was later transported to a laboratory for further analysis. All industrial process steps which were added to PhInd are clearly mentioned in research papers, which is not common, and thus, this type of information is scarce in the literature used for the database. We also highlight that in the majority of the references used for the database, the raw material was entirely generated at the laboratory level (>50%), rather than on the industrial scale; therefore, “Scale” was also added as another search option. This option is particularly useful for users as it separates information about polyphenol content in agri-food by-products made either in controlled laboratory settings or in real-world industrial scenarios. The focus on industrial processes makes PhInd particularly useful for researchers and professionals in fields such as food science, agriculture and biotechnology, and this functionality enables users to explore papers related to specific industries or processes within the plant processing domain.

The third section is associated with the pre-treatment of the raw material prior to the isolation of bioactive compounds. In some cases, the by-product was not subjected to any pre-treatments in the laboratory (e.g., drying, defatting, enzyme pretreatment, etc.) prior to extraction, and this option could be searched for in the first checkbox “Sample” by clicking on the “Non-treated” option. Further pre-treatment checkboxes are associated with laboratory operations performed before the polyphenol extraction step, titled “Defatting” (e.g., organic solvent extraction, supercritical fluid extraction, etc.), “Drying” (e.g., oven, shade, freeze-drying, etc.) and “Thermal/non thermal”, the last being associated mainly for microbial inactivation, conservation of the raw material and physical separation of plant fractions (e.g., pasteurization, roasting, vinification, pulsed electric fields, pressing, etc.). “Hydrolysis” was added as the last pre-treating step in order to highlight whether hydrolysis of bound polyphenols occurred in pre-treatment and/or prior to the HPLC analysis as the extraction step. We pointed this out as an important discriminant since hydrolysis could dramatically alter the chemical profile and polyphenol content in analyzed extracts. For database entries in which the solvent was only mildly acidified (e.g., 0.1% *v*/*v* acetic acid or hydrochloric acid), it was considered that a hydrolysis process was not applied [40,41]. In addition to the term “All”, checkboxes related to pre-treatment have the term “No”, which was marked if a specific manuscript sample was not defatted and/or dried and/or thermally/non-thermally treated and/or hydrolyzed. For example, apple pulp was dried in sunless storage (selected term—shade drying), while defatting, thermal/non-thermal operations and hydrolysis were not performed (selected term “No”) [42].

The last section in the search options is “Extraction”, which should provide information about the applied extraction technique and solvent used for the isolation of polyphenols. Both “Technique” (e.g., solid–liquid extraction, pressurized-liquid extraction, microwave-assisted extraction, etc.) and “Solvent” (e.g., ethanol, acetone, aqueous methanol, water, etc.) are very diverse subgroups and not all cases available in the literature could be added to the checkboxes. We considered that it would be more beneficial to select the major cases in order to narrow down the options. For example, “Solid-liquid extraction” covers various subcases such as maceration, percolation, extraction with mechanical stirring, etc. [43,44]. There are manuscripts that compared two or more different extraction techniques and only the techniques with the highest polyphenolic content (HPLC and/or spectrophotometry) were included in PhInd. For example, only polyphenol content in artichoke leaves obtained by ultrasound-assisted extraction was included in the database due to higher total phenols content. Meanwhile, polyphenol content obtained by using hot water and European Pharmacopoeia extraction protocols were not included due to lower phenolic content [45]. Also, from manuscripts dealing with extraction optimization, only polyphenol content obtained in optimal conditions were added in PhInd, e.g., [46]. There are also some specific cases in which extraction was not performed, e.g., wastewater from olive processing [6] or wastewater remaining after artichoke blanching [7] which contains polyphenols. As for pre-treatment checkboxes, the term “No” was selected for “Extraction” in these cases. Similarly, several solvents given in the checkbox “Solvent” are identified as the most abundant, while less bountiful cases and experimental trials with three or more different solvents were included in PhInd as “Mixture”. Similar to extraction techniques, if different solvents were tested, as it is often the case with deep eutectic solvents (DES) or natural deep eutectic solvents (NADES), only experimental trials with the highest polyphenol content were added in PhInd, e.g., [47].

### 3.2. Additional Options and PhInd Exploration

**Figure 2:**
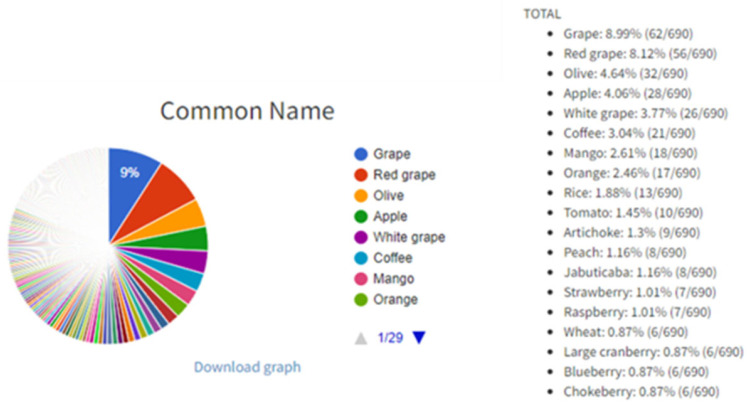
Pie chart of “Common Name” on the graph page displayed as a result of a search.

The buttons “Or” and “And” exist in ‘open’ fields for more tailored searches. For example, it is possible to type “grape mango” in ‘open’ field “Common name” and to select the “Or” button which will, after clicking the “Search” button (magnifier icon in right bottom of the interface), list all entries with grape and mango as a result search. On the other hand, it is possible to type “gallic acid quercetin” in ‘open’ field “Compound(s)” and to select the “And” button in order to list all entries with gallic acid and quercetin. Users can browse the PhInd database by typing in any of the ‘open’ fields and/or selecting one or more checkboxes, and by selecting the “Search” button (Figure 1). On the right-hand side of this button, two other buttons (“Export” and “Graph”) can be used in order to (1) export the search results in an Excel table and (2) obtain pie charts with statistical data including the contribution of each aforementioned characteristic within the performed search results (Figure 2), respectively. Visually presented search results of ‘open’ fields (“Common Name” and “Cultivar/variety”) and all checkboxes are on the “Graph” page. Each graph consists of a colored pie chart in which each term (e.g., grape and red grape) is differently colored and terms are organized by decreasing distribution ratio starting from the term with the highest distribution ratio (e.g., grape 8.99%). The distribution ratio is based on the frequency of appearance of each term in the search results and it is expressed as a percent or total number in parenthesis. For example, if all ‘open’ fields are left empty and checkboxes are selected as “All”, grape appears in 8.99% of entries or 62 out of 690 entries as a result of searching in the PhInd database (Figure 2). Normally, if search criteria are narrower and more specific, pie charts would be different and customized for that specific combination of search criteria. All graph pies can be saved on the user’s PC by clicking “Download graph”. The downloaded file needs only to be “Copy/Paste(d)” to Microsoft Word or Microsoft PowerPoint to be further used as such.

Even though the visual presentation of “Compound(s)” search results would be interesting, since it would display the distribution ratio of individual phenolic compounds in groups of selected entries, this was not technically feasible due to a large number of compounds added to PhInd.

**Figure 3:**
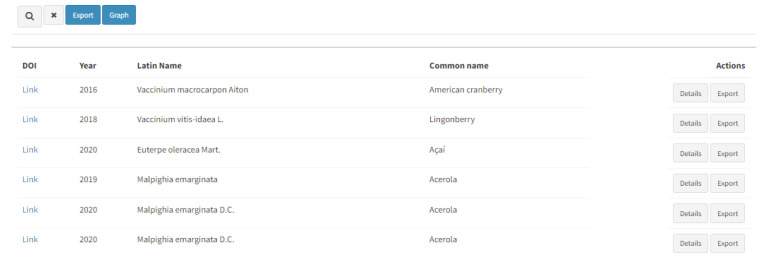
Listed search results with further options for actions (“Details” and “Export”).

By performing the search, the results are given below as a DOI with a hyperlink, year of the publication, Latin name and common name of the resource, and further actions should be performed to reach the desirable information (Figure 3). Two buttons on the right-hand side of each result (“Details” and “Export”) provide thorough information about the entries.

**Figure 4:**
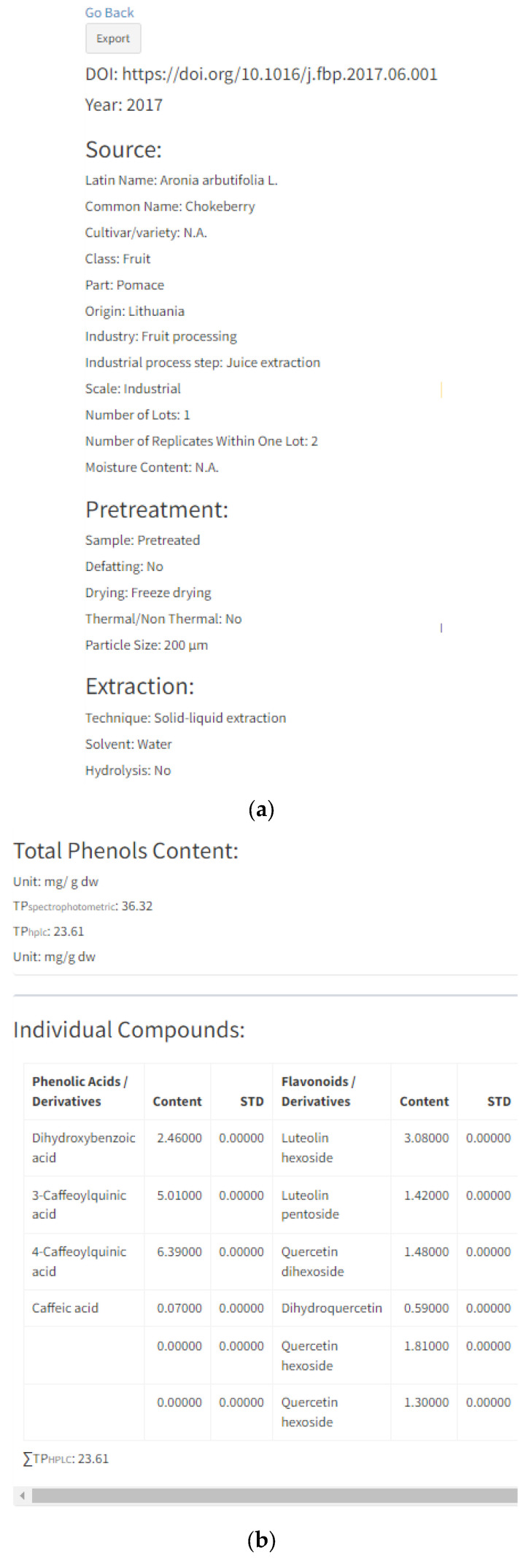

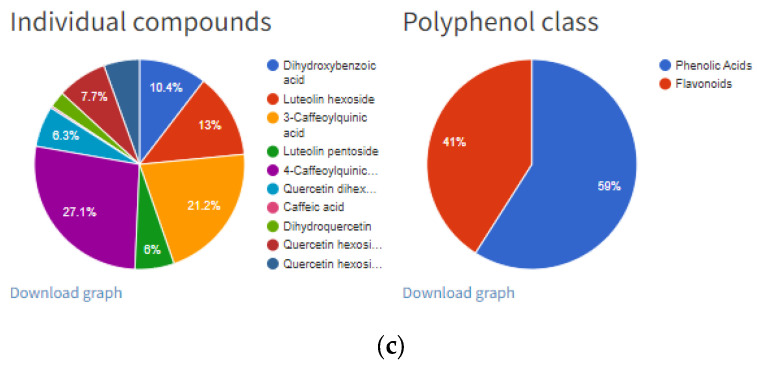
“Details” option of individual entry: (**a**) information related to manuscript identity (DOI and year of publication), plant source, sample pre-treatment and extraction process; (**b**) information related to total phenolic content analyzed with spectrophotometer (TP spectrophotometr) and HPLC (TPhplc), their units and HPLC content of each individual polyphenol; (**c**) chart pie graphs of individual compounds data and polyphenols classes.

By selecting “Details” of each individual entry, a new page will open that contains all information regarding the specific reference, including previously mentioned characteristics given in search options (plant material properties, source properties related to industrial processing, pre-treatment and extraction) (Figure 4a). Information related to the origin, number of lots, number of replicates, moisture content and particle size are only displayed in the “Details” option of each entry. Origin stands for the country of plant cultivation, e.g., wild bilberries from Boyacá region in the Colombian region [48]; the country of company or research institution providing a sample, e.g., raspberry seeds supplied by a Serbian company [49]; or if the prior two are not reported in the manuscript, as the country of the researchers, e.g., Polish researchers working with raspberry and cranberry pomaces [50]. The number of lots stands for a number of different sampling batches, and usually, by-products are from one batch. However, there are some cases with two or more lots which increase data representability, e.g., acerola and jabuticaba pomaces were samples from four different batches of five hundred grams [36]. The number of replicates is a number of HPLC analysis replicates and it is usually three. Moisture content (MC) stands for water content in samples, while particle size (PS) is the interval or average size of the sample particles. Both terms are expressed in the units given in the manuscripts. MS and PS are added since these factors significantly influence polyphenol extraction yield.

**Figure 5:**
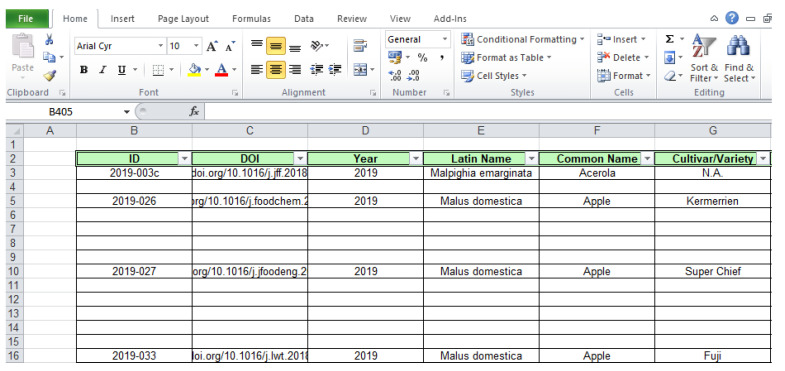
Excel file generated with “Export” option.

Further information will include “Total Phenols Content” and the content of individual polyphenols divided into four subgroups (Figure 4b): (1) phenolic acids/derivatives (e.g., gallic acid, hydroxycinnamic acid derivative, sinapic acid, 5-caffeoylquinic acid, etc.), (2) flavonoids/derivatives (e.g., epicatechin, procyanidin B1, rutin, quercetin, cyanidin-3-O-galactoside, etc.), (3) stilbenes/derivatives (e.g.,resveratrol, cis-piceid, pallidol, etc.), (4) lignans (e.g., lariciresinol, pinoresinol, secoisolariciresinol, etc.) and (5) other (e.g., albiflorin, tyrosol, scopoletin, phloretin, phloridzin, etc.). Total phenol content (TP) was expressed in the units given in their respective manuscripts. All inserted references contain total phenol content expressed as a sum of individual polyphenolic compounds (TPhplc), while total phenol content determined by spectrophotometric assay (TP spectrophotometry) was added where applicable. Polyphenol data inserted in each entry (Figure 4b) are in correspondence with the pie chart graph presented in Figure 4c. These two graphs can be downloaded for each entry and can be further used in Microsoft Word or PowerPoint software. Furthermore, the same results can be generated as an Excel file for each reference by clicking the button “Export” on the right-hand side of the respective reference (Figure 5).

## 4. Conclusions

PhInd is a comprehensive database on polyphenol content (>6000 phenolic composition data) in plant based by-products from the agri-food sector which was developed using peer-reviewed full-text papers (>450 scientific publications). The database was developed with a web interface that should provide a user-friendly search based on one or multiple criteria which could be set using ‘open’ fields or checkboxes. It is possible to search with multiple criteria in four different subsections which are related to (a) plant material properties, compounds and manuscript; (b) source properties related to industrial processing; (c) pre-treatment of the by-product prior to the isolation of bioactive compounds; and (d) the extraction step applied for the isolation of polyphenols. This will allow users to customize their search results in a wide number of different combinations. The results of the search could be explored in detail directly on the website for each entry by clicking the “Details” option or by downloading an Excel file by selecting the “Export” option. This later enables users to obtain full information which could be easily manipulated via the PC “Copy/Paste” option and further used in Microsoft Word or PowerPoint (Windows, Android, macOs and iOS operating systems). At the end, a visual presentation given as downloadable pie graphs enables users to have a quick overview of the data distribution of the search results within selected multiple criteria (e.g., percentage of solid–liquid extraction among other extraction techniques within the selected multiple criteria).

PhInd should be further explored by analyzing the distribution of, e.g., applied extraction techniques, solvents, the type of plant class or part, or other criteria which could provide insights about, e.g., under-utilized plants or under-utilized extraction techniques. This could potentially illuminate the path for further development in scientific areas related to the recovery of polyphenols from plant matrices. Also, industry stakeholders could benefit from such analysis as it could provide information on which extraction techniques or solvents are frequently used to extract polyphenols, which industry generates by-products containing specific polyphenols, etc. Even though PhInd could be a useful tool for a wide range of stakeholders, it has certain limits. Information on polyphenol content in certain agri-food waste by-products can serve only as preliminary guidance on whether certain agri-food by-products can be used as raw material for polyphenol recovery at the industrial level. However, database information cannot replace a thorough chemical analysis because there are so many variables regarding the content of polyphenols in food and therefore in waste products. The addition of data from newly published manuscripts is still not automated and PhInd might not include all relevant plant sources or extraction techniques in the future years. Data quality was rigorously checked by a double-verification process; however, unintentional human errors might have led to some minor errors in polyphenol data since the addition of data was performed manually by a group of scientists.

## Data Availability

All the data is contained within the article.
